# Inhibition of the Prostaglandin Transporter PGT Lowers Blood Pressure in Hypertensive Rats and Mice

**DOI:** 10.1371/journal.pone.0131735

**Published:** 2015-06-29

**Authors:** Yuling Chi, Jean-Francois Jasmin, Yoshinori Seki, Michael P. Lisanti, Maureen J. Charron, David J. Lefer, Victor L. Schuster

**Affiliations:** 1 Department of Medicine, Albert Einstein College of Medicine, Bronx, NY, United States of America; 2 Department of Pharmaceutical Sciences, University of the Sciences in Philadelphia, Philadelphia, PA, United States of America; 3 Department of Biochemistry, Albert Einstein College of Medicine, Bronx, NY, United States of America; 4 Institute of Cancer Sciences, University of Manchester, Manchester, United Kingdom; 5 Department of Obstetrics & Gynecology and Women's Health, Albert Einstein College of Medicine, Bronx, NY, United States of America; 6 Department of Physiology, Louisiana State University Health Sciences Center, Shreveport, LA, United States of America; 7 Department of Physiology & Biophysics, Albert Einstein College of Medicine, Bronx, NY, United States of America; University of Utah School of Medicine, UNITED STATES

## Abstract

Inhibiting the synthesis of endogenous prostaglandins with nonsteroidal anti-inflammatory drugs exacerbates arterial hypertension. We hypothesized that the converse, i.e., raising the level of endogenous prostaglandins, might have anti-hypertensive effects. To accomplish this, we focused on inhibiting the prostaglandin transporter PGT (SLCO2A1), which is the obligatory first step in the inactivation of several common PGs. We first examined the role of PGT in controlling arterial blood pressure blood pressure using anesthetized rats. The high-affinity PGT inhibitor T26A sensitized the ability of exogenous PGE_2_ to lower blood pressure, confirming both inhibition of PGT by T26A and the vasodepressor action of PGE_2_ T26A administered alone to anesthetized rats dose-dependently lowered blood pressure, and did so to a greater degree in spontaneously hypertensive rats than in Wistar-Kyoto control rats. In mice, T26A added chronically to the drinking water increased the urinary excretion and plasma concentration of PGE_2_ over several days, confirming that T26A is orally active in antagonizing PGT. T26A given orally to hypertensive mice normalized blood pressure. T26A increased urinary sodium excretion in mice and, when added to the medium bathing isolated mouse aortas, T26A increased the net release of PGE_2_ induced by arachidonic acid, inhibited serotonin-induced vasoconstriction, and potentiated vasodilation induced by exogenous PGE_2_. We conclude that pharmacologically inhibiting PGT-mediated prostaglandin metabolism lowers blood pressure, probably by prostaglandin-induced natriuresis and vasodilation. PGT is a novel therapeutic target for treating hypertension.

## Introduction

Prostaglandins (PGs) are 20-carbon fatty acids that signal a broad array of physiological events [1]. PGs are synthesized in a series of steps, beginning with the action of cyclooxygenase-1 (Cox-1) or cyclooxygenase -2 (Cox-2) on arachidonic acid to yield PGH_2_. Specific synthases subsequently generate five types of PGs, namely PGE_2_, PGF_2**α**_, PGI_2_, PGD_2_, and thromboxane A_2_ [2]. Synthesis of all five PGs is blocked by either Cox-1/Cox-2 non-selective inhibitors, the so-called non-steroidal anti-inflammatory drugs (NSAIDs), or by the Cox-2 selective inhibitors, known as coxibs [2].

Although normotensive animals and humans generally experience no change in arterial blood pressure (BP) when administered NSAIDs or coxibs, hypertensive rodents and humans exhibit a further rise in BP when given these agents [3–17]. These data suggest that the aggregate effect of endogenous PGs in hypertension is to lower BP toward normal.

If lowering PGs raises BP in hypertension, then increasing PGs may lower BP in hypertension. One approach to increasing PG levels would be to inhibit their metabolism. PGE_2_, PGF_2**α**_, PGD_2_, and PGI_2_, but not thromboxane A_2_, are metabolized by the prostaglandin transporter PGT (SLCO2A1), which is obligatory for PG inactivation [18–22]. PGT-mediated PG uptake across the plasma membrane is followed by cytoplasmic enzymatic inactivation [23]. As predicted from this model, global knockout of PGT gene expression in mice results in elevated systemic levels of the representative prostanoid PGE_2_, and in reduced levels of PGE_2_ metabolite [24]. Similarly, pharmacological inhibition of PGT using a small-molecule inhibitor prevents the catabolism of both endogenous and exogenous PGE_2_ *in vivo* [25].

Based on these findings, we tested the hypothesis that raising systemic PG levels by inhibiting PGT reduces BP in animal models of hypertension.

## Materials and Methods

### Animals

Sprague-Dawley, Wistar-Kyoto, and Spontaneously Hypertensive Rats (SHRs) were purchased from Charles River, Wilmington, MA. C57BL/6 mice, as well as genetically hypertensive mice (BPH/2J) and their matched normotensive mice (BPN/3J), were obtained from the Jackson Laboratory. All experimental procedures done with animals were approved by the Institutional Care and Use Committee at Albert Einstein College of Medicine in accordance with the “Guide for the Care and Use of Laboratory Animals” published by the National Institute of Health.

### Measurement of Arterial Blood Pressure and T26A Half-Life in Anesthetized Rats

Rats weighing 300–350 g were anesthetized with xylazine (10 mg/kg)-ketamine (50 mg/kg) followed by 2000 U heparin (Sigma-Aldrich, St-Louis, MO). After stable anesthesia was obtained, the right jugular vein was isolated and incised, and a polyethylene catheter (PE 50; 0.97 mm OK, 0.58 mm ID) was advanced and positioned in the right ventricle for compound administration. The right carotid artery was isolated and incised, and a millar catheter (SPR-249, Millar Instruments, Houston, TX) was advanced and positioned just above the aortic valve for hemodynamic measurements. The systolic, diastolic, and mean arterial pressures were measured and recorded with the Ponemah P3-Data acquisition system (LDS Test and Measurement, Middleton, WI). BP was immediately recorded after each injection of 100 μL of PGE_2_, or of vehicle (2% DMSO + 2% cremophor for T26A) or T26A, into the jugular vein. Mean BP reduction by PGE_2_ is presented as a percentage = 100 x [(minimum BP immediately after PGE_2_ injection)—(BP immediately before PGE_2_ injection)]/(BP immediately before PGE_2_ injection). Mean BP reduction by T26A is presented as a percentage = 100 x [(minimum BP immediately after T26A or vehicle injection)—(BP immediately before injection)]/(BP immediately before injection).

### Metabolic Recording: Sodium and PGE_2_ Measurements

C57BL/6 mice were placed in metabolic cages and equilibrated on regular water and food for 5 days. Thereafter, they were divided into two groups. One group was put on vehicle (2% DMSO + 2% cremophor in the drinking water), and the other was put on 2 mM T26A in the drinking water. Mice were kept in the metabolic cages for another 20 days. Body weight, food intake, liquid intake, and urine volume were recorded daily. Urines were also collected for determination of urinary volume, [PGE_2_], and [Na^+^]. Na^+^ concentration in urine was measured by an electrode purchased from Lazar Research Laboratories, Inc., Los Angeles, CA. On day 25 at the end of these experiments, blood was withdrawn from each mouse by cardiac puncture and plasma was collected after centrifugation. [PGE_2_] in urine and plasma were measured using EIA kits from Cayman Chemical (Ann Arbor, MI).

### Long-term BP Measurements in Awake, Unrestrained Mice

Genetically hypertensive mice (BPH/2J) or their matched normotensive mice (BPN/3J) at 12 weeks of age were surgically implanted with PA-C10 radiotelemeter pressure transducers (DSI, St. Paul, MN) in the aorta as previously described [26]. After recovery from surgery, mice were first equilibrated with regular water and food for 4–6 days to obtain stable baselines, and then switched to either vehicle (2% DMSO + 2% cremophor) or 2 mM T26A in the drinking water. Aortic blood pressures (systolic, diastolic, and mean) in the conscious, unrestrained state were recorded every 3 hours by radiotelemetry.

### Measurement of Relaxation or Tension of Mouse Aorta

Mouse aortas were harvested from C57BL/6 animals, cleaned, and cut into rings of 2 mm length. Rings were mounted to a myograph system (Danish Myo Technology A/S, Aarhus, Denmark) and bathed with 5 mL oxygenated and warmed (37°C) physiological salt solution (PSS, mM: NaCl 130, KCl 4.7, KHPO_4_ 1.18, MgSO_4_ 1.17, CaCl_2_ 1.6, NaHCO_3_ 14.9, dextrose 5.5, and CaNa_2_ EDTA 0.03, 95% O_2_/5% CO_2_). Rings were set at 700 mg passive tension and were then equilibrated for 1 hour and washed every 20 minutes with PSS. Thereafter, various concentrations of T26A (0, 0.2, 1, and 5 μM) were added in 4 separate baths. 5 minutes after incubation with T26A, the rings were contracted with 1 μM serotonin (5-HT). When the contraction reached a plateau, 10 nM PGE_2_ was added to induce relaxation. Relaxation from PGE_2_ is presented as a percentage = 100 x [(tension immediately before PGE_2_ injection)—(minimum tension immediately after PGE_2_ injection)]/(tension immediately before PGE_2_ injection).

### PGE2 Measurements in Aorta Buffer

About 20 mg of aortas isolated from mice were incubated in 250 μL buffer (PSS, mM: NaCl 130, KCl 4.7, KHPO_4_ 1.18, MgSO_4_ 1.17, CaCl_2_ 1.6, NaHCO_3_ 14.9, dextrose 5.5, and CaNa_2_ EDTA 0.03) in a 1.5-mL Eppendorf tube, after which 2 μL of either vehicle (ethanol) or 1 mM T26A was added to the aorta incubation buffer. The final concentration of T26A was 10 μM. At 10 minutes after T26A/vehicle addition, 100 ng arachidonic acid (AA) (Cayman Chemical, Ann Arbor, MI) in 2.5 μL ethanol was added to each tube and aortas were incubated for another 3 or 5 minutes. Then the buffer was collected for PGE_2_ measurements using the EIA kits from Cayman Chemical.

### Statistical methods

Differences between data sets were analyzed by two-tailed paired or unpaired t-test as appropriate with a cut-off value for rejection of the null hypothesis of p < 0.05.

## Results

### Intravenous T26A Potentiates the Effect of PGE_2_ on BP in Anesthetized Sprague-Dawley Rats

**Fig 1:**
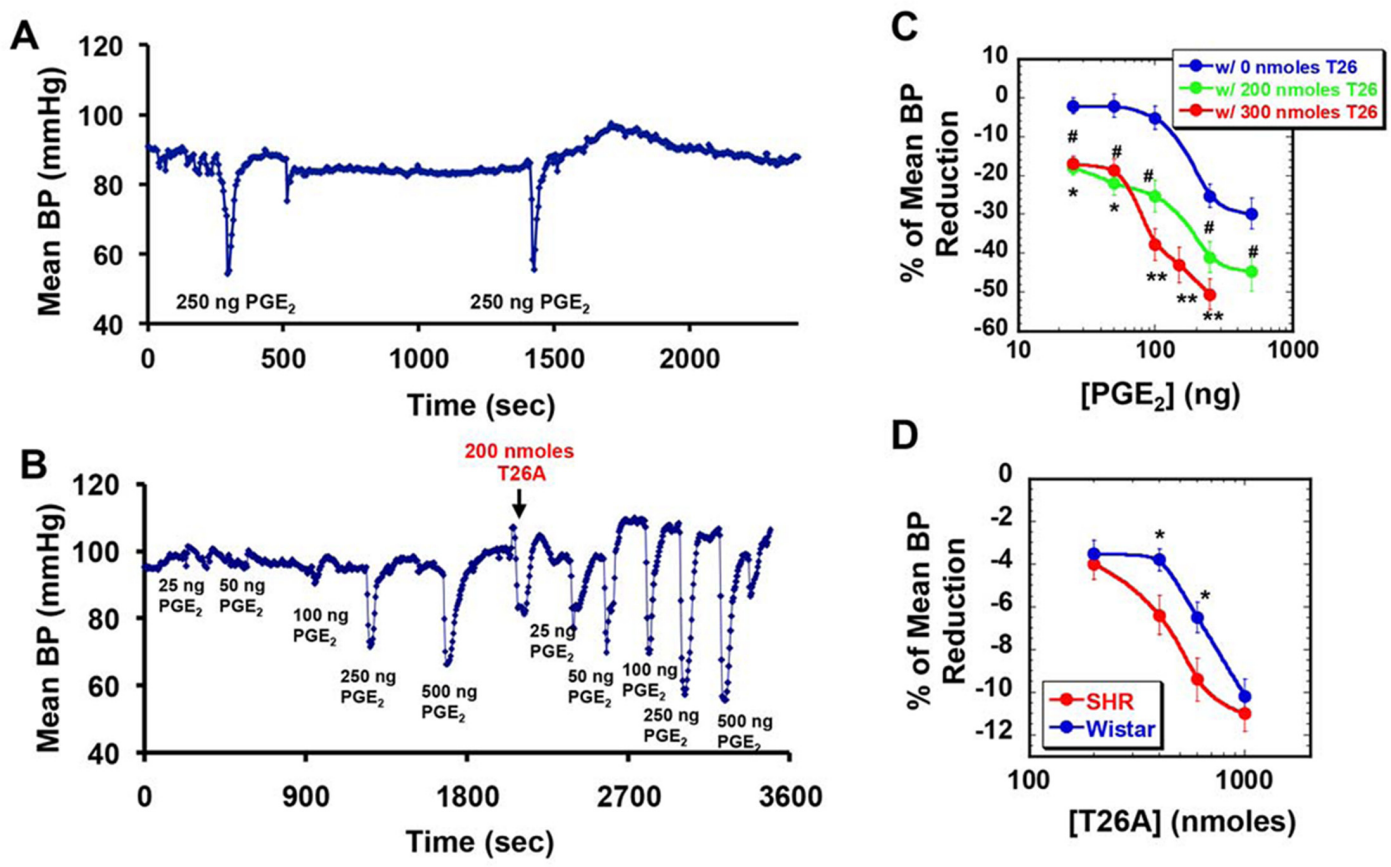
Intravenous T26A dose-dependently potentiates the hypotensive effect of intravenous PGE_2_ in anesthetized normotensive rats, and lowers BP in hypertensive > normotensive rats. Anesthetized rats were injected intravenously via the femoral vein and arterial BP was monitored continuously via the carotid artery. **A,** BP of a Sprague-Dawley rat after two intravenous PGE_**2**_ injections at the same dose (250 ng). The reduction in BP is highly reproducible and non-additive, indicating that the first dose of PGE_**2**_ has been rapidly metabolized. **B,** BP of a Sprague-Dawley rat measured after each of a series of stepwise intravenous PGE_**2**_ injections (25–500 ng) before and after T26A injection. T26A potentiates the hypotensive effect of each does of PGE_**2**_. **C,** Summary of a series of studies performed as in panel B, after injecting 0 nmoles, 200 nmoles, or 300 nmoles of T26A in the second phase. Percent reduction of BP was calculated as per Methods. T26A shifted the dose-response curve of PGE_**2**_ on BP to the left in a dose-dependent fashion. Values are mean ± SEM, n = 5 rats for each point. # p < 0.05 for 200 nmoles versus 0 nmoles T26A. * p < 0.05 and * p < 0.01 for 300 nmoles versus 0 nmoles T26A. **D,** Effect of intravenous T26A alone on BP of normotensive Wistar-Kyoto rats (blue) and SHR rats (red). Percent reduction of BP was calculated as per Methods. T26A produced a greater fractional reduction in BP in SHR versus Wistar-Kyoto rats. Mean ± SEM, n = 5 rats each. * p < 0.05.

We had previously shown that intravenous T26A increases the concentration of arterial PGE_2_ in normotensive, anesthetized rats [25]. Because some PGE_2_ receptors are vasodepressor and some are vasoconstrictor [27], we extended this earlier determination of [PGE_2_] only to now comprise BP measurements. Using normotensive Sprague-Dawley rats, we first injected T26A or vehicle intravenously, and then injected PGE_2_ and measured the BP response at the right carotid artery. As shown in Fig 1A, a 250 ng intravenous bolus of PGE_2_ induced a transient reduction in arterial BP, an effect that was highly reproducible, since a subsequent injection about 20 minutes later of 250 ng of PGE_2_ caused the same degree of BP reduction without any cumulative effect. This result is consistent with the known rapid metabolic inactivation of PGE_2_ by PGT [28].

Using a similar protocol, we pre-loaded rats with 300 or 500 nmoles of intravenous T26A and then probed the arterial BP responses to three sequential 100 ng boluses of PGE_2_ over the subsequent 50 minutes. These experiments yielded an apparent dynamic half-life for T26A of 40 and 50 minutes, respectively, for the two doses.

In a PGE_2_ dose-response protocol (Fig 1B, left portion), incrementally larger doses of intravenous PGE_2_ caused dose-dependent transient reductions in arterial BP, with a return to baseline BP between the reductions. We then injected 200 or 300 nmoles of T26A. As shown in Fig 1B, this produced a transient drop in BP, followed by an overshoot and then a return to baseline. The overshoot likely indicates activation of reflex vasoconstrictor tone in response to vasodilator effects (PG-mediated) of T26A.

Repeating the PGE_2_ ramp following T26A injection resulted in a significantly augmented effect of any given dose of intravenous PGE_2_ on BP (Fig 1B, right portion). The baseline BP drifted upwards at the highest doses of PGE_2_ post-T26A, likely representing additional activation of reflex vasoconstrictor tone. It is important to note that the extreme right-hand tracing of Fig 1B represents significant challenges to BP homeostasis by large boluses of vasodilator PGE_2_ in the presence of blocked PGE_2_ metabolism. These conditions do not reflect a normal physiological state, thus the upward drift in baseline BP at the highest PGE_2_ doses does not constitute a negation of the use of pharmacological PGT inhibition for hypertension.

These experiments are summarized in Fig 1C, which demonstrates that T26A significantly shifted the PGE_2_ dose response curve to the left, and also that the extent of the left shift is dependent on the dose of T26A.

Taken together, these studies indicate that PGE_2_ is a vasodepressor in the rat and that PGT activity modulates the ability of PGE_2_ to reduce BP.

### Intravenous T26A Reduces Arterial Blood Pressure to a Greater Degree in Anesthetized SHR Rats than in Wistar-Kyoto Rats

With the intention to evaluate T26A as a prototype antihypertensive, and knowing that PGT inactivates PGs other than PGE_2_ [18–22], we measured the effect of intravenous T26A alone on BP in anesthetized rats. Inspection of Fig 1B reveals that T26A induced a transient reduction of BP in normotensive rats. To examine this effect further, we injected various doses of T26A intravenously into both normotensive (Wistar-Kyoto) and hypertensive (SHR) anesthetized rats. As shown in Fig 1D, T26A over the range of 200 to 1000 nmoles (0.15 to 0.75 mg per 300 g rat) dose-dependently reduced arterial BP in both normotensive Wistar-Kyoto and hypertensive SHR rats, with a larger fractional BP reduction in the hypertensive animals. At the maximal dose of 1000 nmoles of T26A, the absolute magnitude change was equivalent for Wistar-Kyoto and SHR. In the absence of detailed binding studies that are beyond the scope of the present investigation, we speculate that at these doses T26A may saturate PGT binding sites, eliciting a maximal effect on BP reduction.

### Oral Administration of PGT Inhibitor Raises Endogenous PGE_2_ Levels, but not Thromboxane Levels, in Awake, Ambulatory Mice

We extended these studies to the oral administration route by administering T26A in the drinking water to awake, ambulatory mice. T26A was first dissolved in pure DMSO at a concentration of 100 mM and then mixed with drinking water at a final concentration of 2 mM. In addition, 2% (v/v) cremophor was added to the mixture to make it homogenous. Vehicle-treated mice received the same 2% DMSO + 2% cremophor in the drinking water but without T26A.

**Fig 2:**
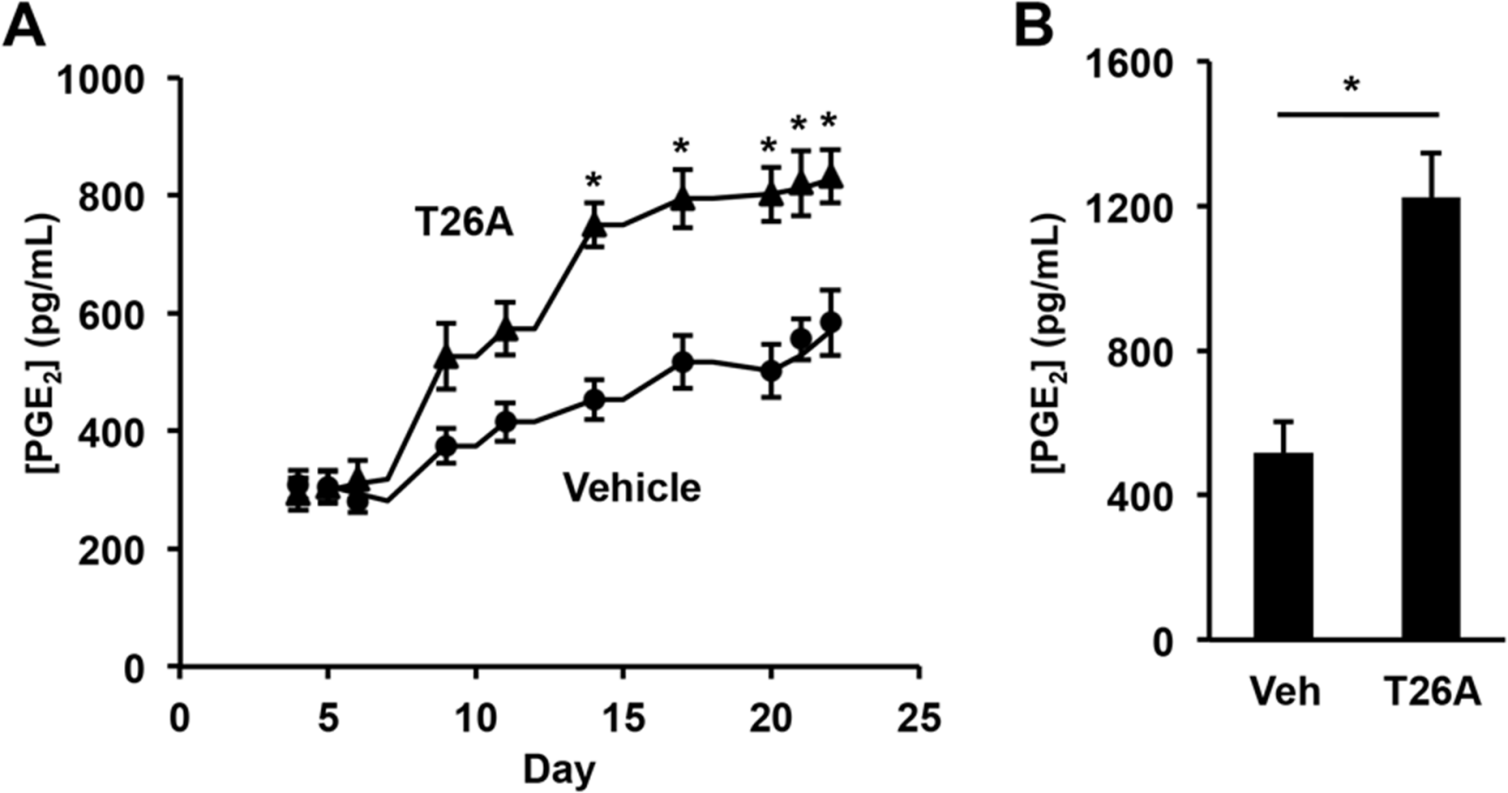
Inhibition of PGT by oral T26A raises urinary and plasma PGE_2_ concentrations in awake, ambulatory mice. Mice were kept in metabolic cages and fed with regular water and food for 5 days. Vehicle (2% DMSO + 2% cremophor) or 2 mM T26A was added to the drinking water on day 6. Urines were collected every 24 hours and averaged as in the text. On day 25, blood was drawn by cardiac puncture. **A,** PGE_**2**_ levels in urine of mice on oral vehicle or T26A. **B,** PGE_**2**_ levels in plasma of mice at the termination of the study. Values are mean ± SEM, n = 6 mice each, *p < 0.05.

T26A-treated mice had urinary PGE_2_ concentrations that increased beginning three days after starting oral T26A and reached a stable level by day 10 (Fig 2A). Although the vehicle necessary to solubilize T26A had its own effects in these and subsequent oral experiments, in all cases the changes seen with T26A were statistically greater than those with vehicle alone. After 3 weeks, the mice were bled, at which point there was a statistically significant 2-fold increase in plasma PGE_2_ concentrations in the T26A-treated mice compared to vehicle-treated mice (Fig 2B). These data are consistent with our previous findings of elevated urinary PGE_2_ levels in PGT knockout mice [24] and validate that oral administration of T26A effectively inhibits PGT *in vivo*.

Measurements of the stable thromboxane A2 metabolite thromboxane B2 (TxB2) on the urine samples showed that T26A had no effect on this prostaglandin (S1 Fig, Inhibition of PGT by oral T26A has no effect on urinary excretion of the thromboxane metabolite TxB2), consistent with the lack of transport of thromboxane by PGT [18]

### Oral T26A Reduces Arterial BP in Hypertensive Awake, Unrestrained Mice

We next asked whether T26A could function as an oral antihypertensive agent in awake, hypertensive animals. We measured BP telemetrically in genetically hypertensive mice (BPH/2J) and their corresponding normotensive mice (BPN/3J) [29]. Radio-telemetry pressure transducers (DSI, Inc., St. Paul, MN) were implanted surgically in the carotid artery of the mice. Surgery was followed by a one-week recovery period during which baseline BP measurements were obtained. Thereafter, T26A or vehicle was added to the drinking water.

**Fig 3:**
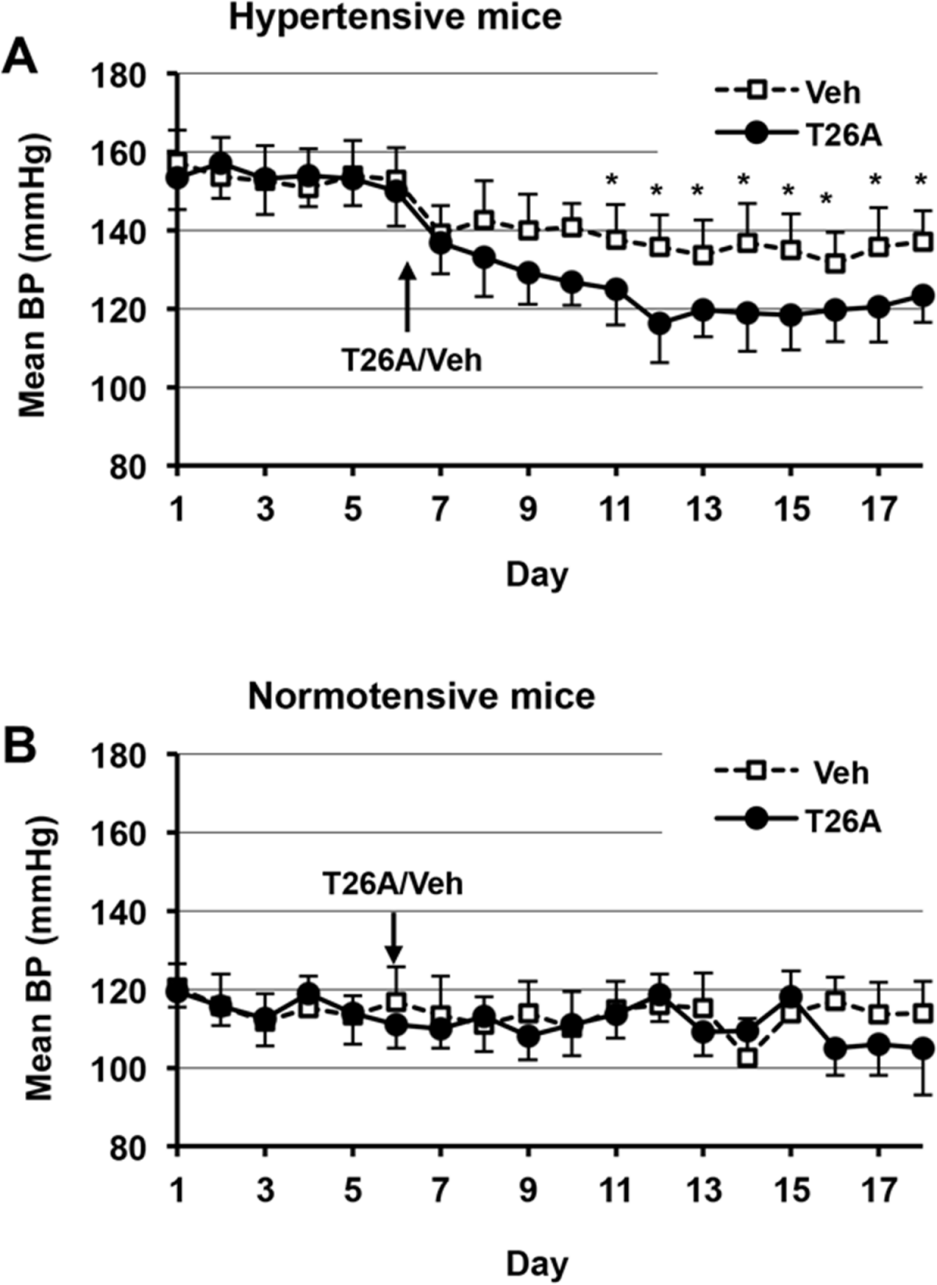
Oral T26A reduces arterial blood pressure (BP) in awake, ambulatory hypertensive mice. Genetically hypertensive mice (BPH/2J) and their normotensive controls (BPN/3J) were instrumented with telemetric BP sensors inserted in the carotid artery. From days 0–5 post-operatively, the mice were given normal chow and drinking water. Starting on day 6, the mice were switched to either vehicle (2% DMSO + 2% cremophor) or 2 mM T26A in the drinking water for an additional 13 days. Arterial BP was monitored radiotelemetrically and recorded every 3 hours. For each mouse, the daily mean BP was taken as the mean of the 8 PB measurements during that day. Values are the average of such daily mean BPs from 4 mice in each group. Bars are SEM. **A,** Effect of oral vehicle or T26A on BP in hypertensive (BPH/2J) mice. **B,** Effect of oral vehicle or T26A on BP in normotensive (BPN/3J) mice. * P < 0.05.

In hypertensive mice with a baseline mean BP of 145 mm Hg, T26A reduced mean BP to 120 mm Hg, with statistical significance against vehicle-treated mice achieved within 5 days (Fig 3A). Of interest, in normotensive mice, T26A did not produce a statistically significant reduction of mean BP (Fig 3B).

### Inhibition of PGT Increases Renal Sodium Excretion in Awake, Unrestrained Mice

**Fig 4:**
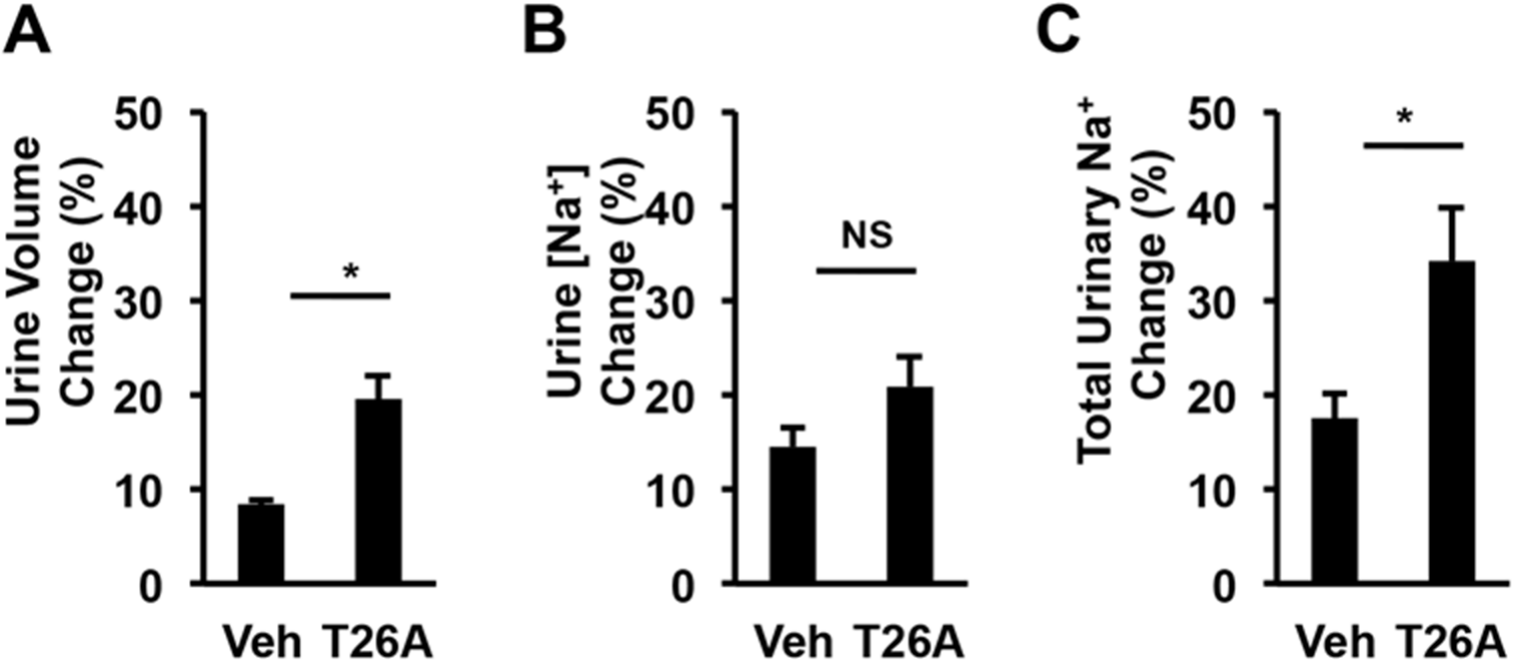
Oral T26A increases urinary water and sodium excretion in awake, ambulatory mice. As in Fig 2, C57BL/6 mice were kept in metabolic cages and fed with regular water and food for 5 days. Vehicle (2% DMSO + 2% cremophor) or 2 mM T26A was added to the drinking water on day 6. Urines were collected every 24 hours. For each mouse, daily urine volume, urinary sodium concentration, and total sodium excretion were calculated as the average of the last three equilibration (control) days and of the first three experimental days. Percent changes were calculated as per Methods. **A,** Percent change in daily urine volume from vehicle or T26A. **B,** Percent change in urinary sodium concentration. **C,** Percent change in total urinary sodium excretion. Values are mean ± SEM, n = 6 mice each, *p < 0.05, NS, not significant.

To test the hypothesis that, by increasing plasma PG levels, orally administered T26A induces renal natriuresis and diuresis, we measured urine volumes and sodium concentrations collected for the experiments shown in Fig 3. We averaged values obtained over the 3 days immediately preceding T26A administration, and also over the first 3 days of oral T26A administration. Fig 4 shows that, compared to vehicle-treated control animals, oral administration of T26A induced a statistically significant 80% increase in daily urine volume (Fig 4A); a non-statistically significant increase in urinary sodium concentration of 35% (Fig 4B); and a statistically significant increase of 100% in daily total sodium output (Fig 4C). Thus, inhibiting PGT with oral T26A induces diuresis and natriuresis.

### Inhibition of PGT Increases Net Release of PGE_2_ from Mouse Aorta and Induces Vascular Relaxation in Mouse Aortic Rings

Using cell culture model systems, we have advanced the hypothesis that PGs are released on demand, bind to and activate nearby receptors, and are then taken back into the cell by PGT for purposes of enzymatic oxidative inactivation [22]. PGT is highly expressed in endothelia [30–33], raising the likelihood that PGT modulates PG inactivation in the vasculature. To address this question, we examined the effect of T26A on the net release of the representative prostaglandin PGE_2_ from isolated mouse aortas following stimulation by arachidonic acid. T26A resulted in a significant increase in the medium PGE_2_ concentration (vehicle: 231 ± 21 SEM pg/mL/mg aorta versus T26A: 391 ± 34 SEM pg/mL/mg aorta, n = 3 aortas each, p = 0.016).

**Fig 5:**
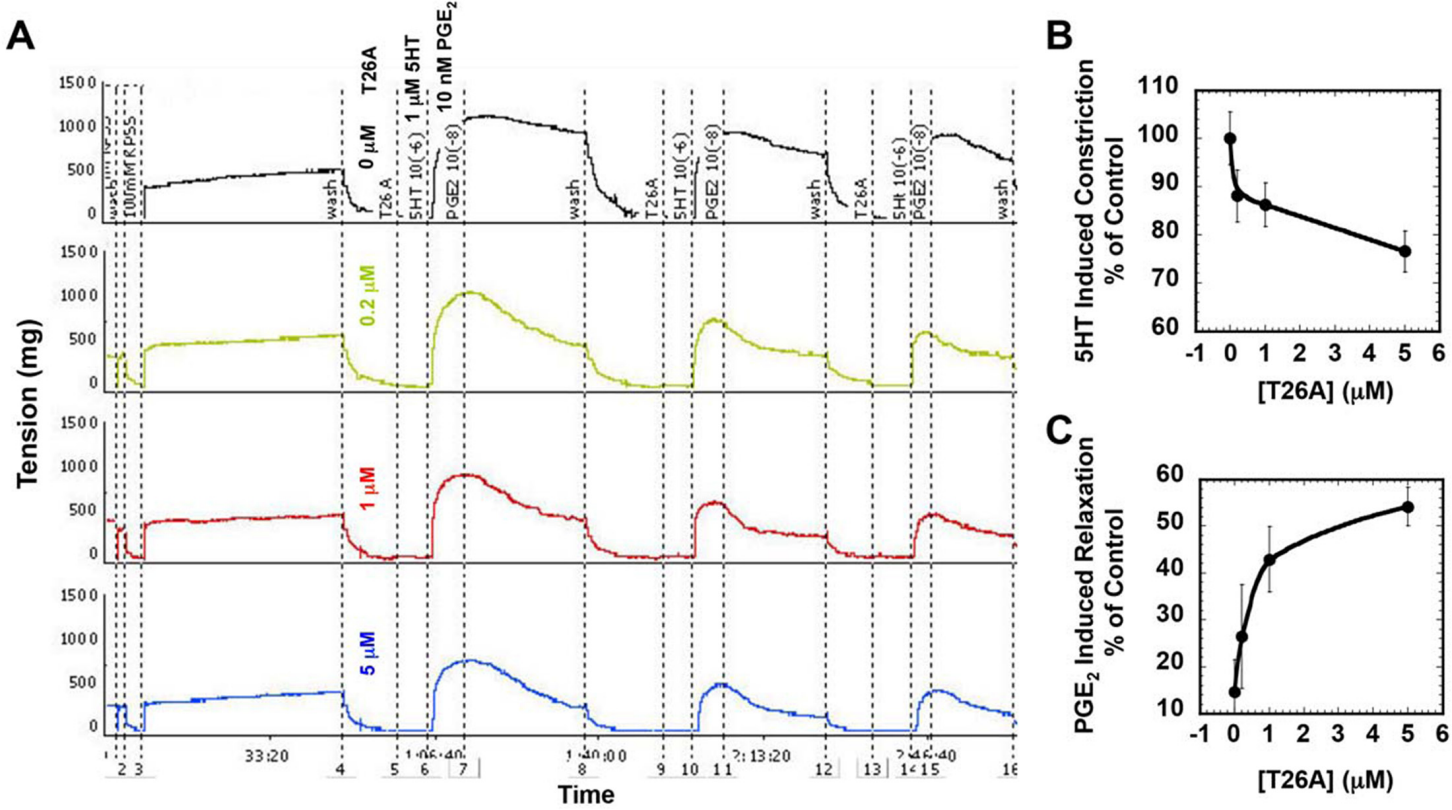
T26A mitigates constriction induced by serotonin, and potentiates relaxation induced by exogenous PGE_2_, in isolated mouse aortic rings. Aortic rings from C57BL/6 mice were mounted on tension transducers in bathing solutions and the tension monitored over time. After equilibration and contraction with 60 mM KCl, and a subsequent wash, 5 μL of T26A at various concentrations in ethanol was added to the incubation buffer. **A,** Representative experiment showing four rings from a single mouse aorta studied simultaneously. The final concentrations of T26A were (top to bottom) 0, (black), 0.2 μM (green), 1 μM (red), and 5 μM (blue). Five minutes after T26A addition, 1 μM 5-hydroxytryptamine (5-HT) was added to induce contraction. When the tension reached a plateau, 10 nM PGE_**2**_ was added to induce relaxation. After the tension again reached stable levels, aortic rings were washed twice for about 20 minutes each, and then the sequential addition of 5-HT and PGE_**2**_ was repeated twice more. **B,** the maximum tension induced by 5-HT (B), and **C,** the maximum relaxation induced by PGE_**2,**_ each calculated as per Methods, were taken from 3 measures for each aortic ring. For each T26A concentration, triplicate experiments were conducted with fresh aortic rings. Values are mean ± SEM, n = 3 mice for each data point. T26A dose-dependently reduced 5-HT-induced vasoconstriction and augmented PGE_**2**_-induced vasorelaxation.

We assessed whether the T26A-induced increase in net PG release has an effect on vascular resistance by measuring the constriction of isolated mouse aortic rings in response to vasoactive agents in the absence or presence of T26A. We pre-treated the rings with vehicle or T26A (0.2, 1.0, or 5 μM), induced aortic contraction with the vasoconstrictor serotonin (5-HT, 1 μM) [34], and then induced vasodilation with 10 nM PGE_2_. Fig 5A shows a representative experiment in which T26A both blunted 5-HT-induced vasoconstriction and also amplified the subsequent PGE_2_-induced vasodilation. Fig 5B shows that, on average, the T26A effect on 5-HT-induced vasoconstriction was dose-dependent, and that 5 μM T26A blocked 25% of 5-HT induced vasoconstriction. Fig 5C shows that T26A also augmented PGE_2_-induced vasodilation in a dose-response manner, such that 5 μM T26A enhanced the relaxation induced by PGE_2_ by over 50%. Thus, PGT directly regulates autocrine control of vasomotor tone by PGs in isolated mouse aortas.

### Chronic Oral T26A is Non-Toxic to Mice

To evaluate further T26A as a prototype antihypertensive agent, we monitored food intake, water intake, and body weight of mice administered T26A in the drinking water for 24 weeks. T26A did not cause any statistically significant abnormalities in any of these parameters (S2 Fig, Oral T26A does not affect food and water intake or body weight). We also conducted necropsies on these mice. S3 Fig and S4 Fig show that T26A caused no histological changes in the heart, lung, kidney, or gastrointestinal tract (S3 Fig, Histology of lung, heart and kidney; S4 Fig, Histology of the intestinal tract).

## Discussion

**Fig 6:**
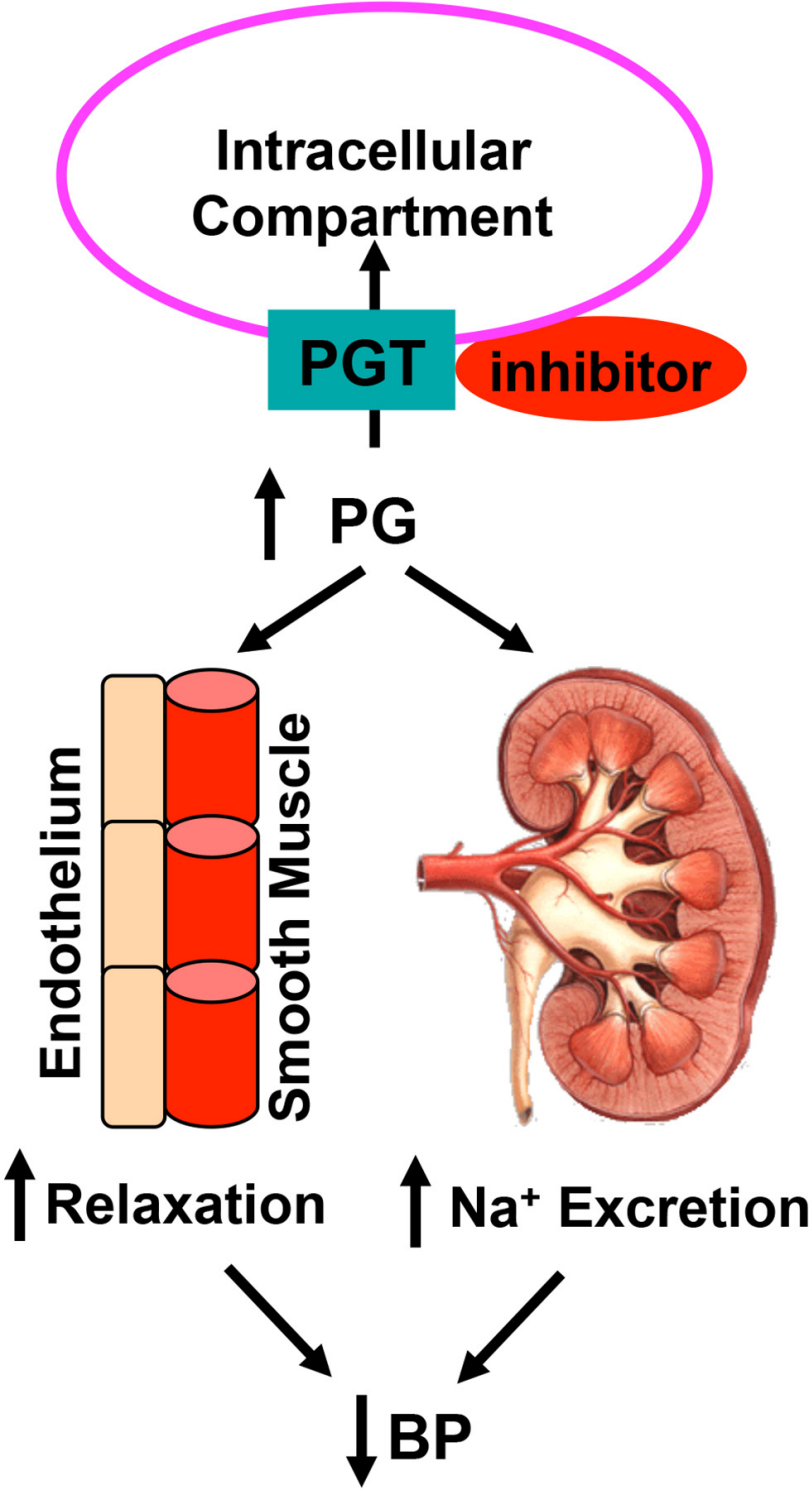
Proposed model by which T26A may reduce BP. Inhibition of PGT-mediated PG reuptake results in increased extracellular PGs. The PGs, in turn, may induce vascular relaxation and stimulate sodium excretion, both of which would reduce BP.

The main findings are that inhibiting the PG transporter PGT with the small-molecule inhibitor T26A lowers BP in hypertensive rats and mice. Additional data suggest at least two mechanisms for this effect. First, in isolated mouse aortas, T26A increases the extracellular concentration of endogenously-formed PGE_2_, consistent with the presence of an autocrine PG loop in this vessel. In these aortas, T26A also inhibits the vasoconstrictive ability of serotonin and augments the vasodepressor activity of exogenously-added PGE_2_. Second, in intact mice, oral T26A increases urinary excretion of PGE_2_, confirming PGT inhibition by the oral route, and it induces natriuresis and diuresis. Together, these data suggest that inhibiting PGT may reduce BP by a combination of vasodilation and enhanced renal sodium excretion (Fig 6).

Pressors induce the release of vasodilatory PGs from the endothelium [35–41]. This autocrine negative feedback loop appears to be operative in hypertension, since reducing PG synthesis raises BP in hypertension [3–17]. PGT is highly expressed at the luminal membrane of endothelia [30–33] and is therefore in an ideal location to modulate the local concentration of vasodilatory PGs. Indeed, inhibiting PGT with T26A increased net PG release by isolated mouse aortas and reduced the vasopressor action of serotonin (Fig 5). We interpret these results to be consistent with a model in which antagonizing PGT augments the autocrine vasodepressor PG feedback loop, at least in this large vessel. These results are in accord with our recent report, using a reconstituted cell culture autocrine signaling system, that PGT controls cell-surface PGE_2_ concentrations arising from peptide hormone stimulation and, as a result, controls PGE_2_ access to, and activation of, autocrine PGE_2_ (EP) receptors [42]. The present results are also consistent with a previous report in which blockade of PGT-mediated PGE_2_ reuptake by a less potent forerunner of T26A [43] resulted in vasodilation of rat brain arterioles [44].

Despite the ability of vasoconstrictors to induce release of vasodepressor PGs in blood vessels, as described above, both hypertensive humans and rodents [45,46], including the SHR model used here [47–54], exhibit impaired production and/or signaling by vasodepressor PGs. In this regard, we found that T26A lowered arterial BP in hypertensive, but not normotensive, mice (Fig 2), and that it induced a proportionally larger reduction in arterial BP in SHR, as compared to normotensive, rats (Fig 1). These results suggest that a relative paucity of vasodepressor PGs in hypertension might be ameliorated, at least in part, by blocking PG metabolism. Additional experiments beyond the scope of the present investigation will be required to address this hypothesis in detail.

It is important to note that the strong vasoconstrictor thromboxane A2 is not transported by PGT [18], and that inhibiting PGT with T26A did not change systemic thromboxane levels as judged by urinary excretion (S1 Fig, Inhibition of PGT by oral T26A has no effect on urinary excretion of the thromboxane metabolite TxB2). On the other hand, PGE_2_, PGF_2**α**_, PGD_2_, and PGI_2_ are all transported by PGT [18–21]. Although only the representative PGE_2_ among these four PGs was measured here, ambient levels of the other three PGs would be expected to similarly increase upon inhibiting PGT. Of the four remaining PGs, only PGF_2**α**_ is vasoconstrictive [55,56].

The remaining PGs transported by PGT are vasodepressors. Although the PGE_2_ receptors EP_1_ and EP_3_ are vasoconstrictive in mice, the EP_2_ and EP_4_ receptors are vasodepressive [27]; experiments using PGE_2_ synthase knockout mice subjected to salt-loading, angiotensin II, or aldosterone-induced hypertension indicate that endogenous PGE_2_ is, in the aggregate, anti-hypertensive [27]; and exogenous PGE_2_ lowers arterial BP through both vasodepressor and natriuretic effects in experimental animals and human subjects [57–64]. PGD_2_ causes either mild vasodilation or has no effect on BP in rats and humans [45,57,65]. Finally, PGI_2_ is an extremely potent vasodilator [66], and mice with disrupted PGI_2_ signaling develop hypertension when salt loaded [67,68]. Thus, in the aggregate it appears that the antihypertensive actions of PGE_2_, PGD_2_, and PGI_2_ prevail over the vasoconstrictive actions of PGF_2**α**_ when PGT is inhibited.

The combination of aortic constriction (Fig 5) and renal diuresis and natriuresis (Fig 4) induced by T26A is of interest. In treating hypertension, vasodilators alone often induce intense renal Na^+^ retention [69,70], whereas diuretics alone often induce intense vasoconstriction [71]. Each of these reciprocal compensations reduces the effectiveness of the primary strategy. The apparently dual action of T26A is reminiscent of that seen with the highly successful angiotensin converting enzyme inhibitors [72–74].

The use of two hypertension animal model systems allowed us to examine changes in BP from T26A that was given via two separate administration routes over two disparate time frames in two distinct rodent species. Moreover, the so-called Schlager BPH/2J mouse is genetically hypertensive primarily from excess sympathetic nervous activity [75], whereas the SHR rat is a normal-renin, relatively sodium independent model in which CNS, neurohumoral, renal, and cellular abnormalities appear to play roles [76]. We found that raising endogenous PGs by inhibiting PGT lowered arterial BP in both of these rodent models (Figs 1 and 3). The rats were studied acutely following intravenous injection, a drug delivery method known to generally yield a sharp plasma peak followed by a trough. It is therefore likely that the T26A-induced decrease in BP in normotensive rats was due to transiently high plasma levels. In contrast, oral delivery, especially in a continuous delivery mode like drinking water, would be predicted to produce lower, albeit more sustained, plasma concentrations, and thus would probably not reach plasma levels sufficient to induce a drop in BP in normotensive animals. Since the etiology of human essential hypertension is multi-factorial, including renal sodium retention and excessive vascular constriction [77,78], our results in these two distinct models of hypertension suggest that PGT inhibition may be broadly applicable to many forms of this disease.

One *a priori* concern with inhibiting PGT in humans might be that the physiological actions of PGs are myriad [79] and therefore the approach might adversely influence multiple physiological pathways. Surprisingly, appropriately rescued PGT knockout mice survive patent ductus arteriosus at birth and appear normal thereafter [24]. Similarly, mice treated for more than 3 weeks with systemic T26A exhibited no visible phenotypic abnormalities (S2 Fig, Oral T26A does not affect food and water intake or body weight; S3 Fig, Histology of lung, heart and kidney; S4 Fig, Histology of the intestinal tract). Humans null at both alleles of the PGT locus exhibit no symptoms until puberty, at which point males, but not females, develop a rather limited phenotype consisting of thickened cephalic skin, digital clubbing, and periosteal calcification [80–88]. These seemingly minor clinical consequences of inhibiting PGT either genetically or pharmacologically in mice or humans support our pursuit of PGT as a prospective drug target.

As of 2010, high blood pressure ranked as the leading single risk factor in the “global burden of disease” analysis [89]. Despite the seemingly large number of antihypertensive agents available, and despite the belief by many clinicians that hypertension can be typically well managed with currently available drugs, in fact the majority of patients with hypertension who are treated with drugs do not attain goal BP levels, and there is an unmet need to find new antihypertensive drugs that are safe, reduce BP effectively, and provide target-organ protection [90]. Aliskiren, the most recent new first-in-class drug for hypertension, was approved seven years ago, and the last new molecule, azilsartan, was approved three years ago. In contrast, in the same time period the FDA has approved five novel anticoagulants (with three different modes of action), four new antiplatelet agents (two different modes of action), and five new molecules for the treatment of pulmonary hypertension (four modes of action) [91]. Here we have provided a demonstration that pharmacologically inhibiting the PG transporter PGT reduces BP by two modes of action in hypertensive mice and rats. PGT bears further exploration as an attractive new drug target in human essential hypertension.

## Supporting Information

S1 Fig Inhibition of PGT by oral T26A has no effect on urinary excretion of the thromboxane metabolite TxB2. (PDF)

S2 Fig Oral T26A does not affect food and water intake or body weight. (PDF)

S3 Fig Histology of lung, heart and kidney. (PDF)

S4 Fig Histology of the intestinal tract. (PDF)
