# Supplementary material for: Inhibition of the Prostaglandin Transporter PGT Lowers Blood Pressure in Hypertensive Rats and Mice
Source: PLoS One. 2015 Jun 29;10(6):e0131735. doi: 10.1371/journal.pone.0131735 (PMC4488299; doi:10.1371/journal.pone.0131735)
Supplement: S1 Fig — (PDF) [file pone.0131735.s001.pdf]

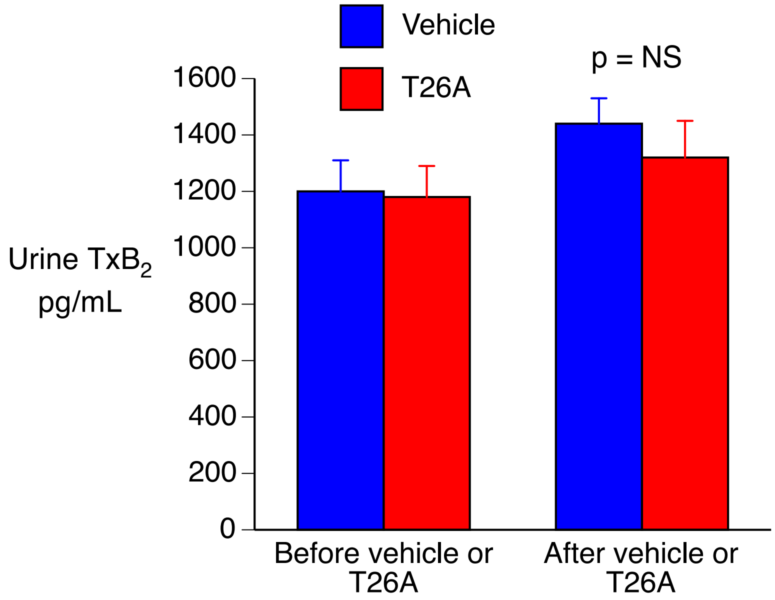

S1 Fig. Inhibition of PGT by oral T26A has no effect on urinary excretion of the thromboxane metabolite TxB2. Mice were kept in metabolic cages and fed with regular water and food for 5 days. Vehicle (2% DMSO + 2% cremophor) or 2 mM T26A was added to the drinking water on day 6. Urines were collected every 24 hours. TxB_2 was measured in urines collected on day 5 (before agent onset) and on day 22 (16 days after agent onset). Values are mean ± s.e.m. (n = 6 mice).
