# Supplementary material for: Inhibition of the Prostaglandin Transporter PGT Lowers Blood Pressure in Hypertensive Rats and Mice
Source: PLoS One. 2015 Jun 29;10(6):e0131735. doi: 10.1371/journal.pone.0131735 (PMC4488299; doi:10.1371/journal.pone.0131735)
Supplement: S2 Fig — (PDF) [file pone.0131735.s002.pdf]

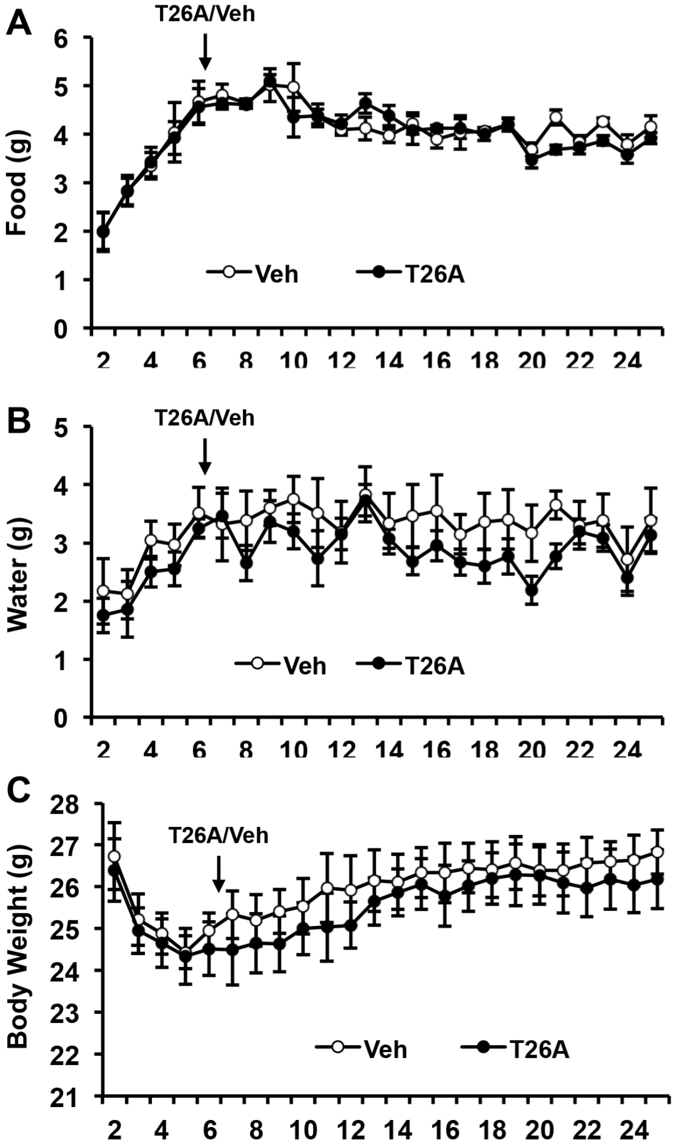

S2 Fig. Oral T26A does not affect food and water intake or body weight. Food (**A**) and water (**B**) intake and body weight (**C**) of mice receiving regular food and water containing vehicle (2% DMSO + 2% cremophor), or 2 mM T26A in the drinking water for 16 days. Values are mean ± SEM (n = 6 mice). None of the points is statistically significant for vehicle versus T26A.
