# Supplementary material for: Inhibition of the Prostaglandin Transporter PGT Lowers Blood Pressure in Hypertensive Rats and Mice
Source: PLoS One. 2015 Jun 29;10(6):e0131735. doi: 10.1371/journal.pone.0131735 (PMC4488299; doi:10.1371/journal.pone.0131735)
Supplement: S3 Fig — (PDF) [file pone.0131735.s003.pdf]

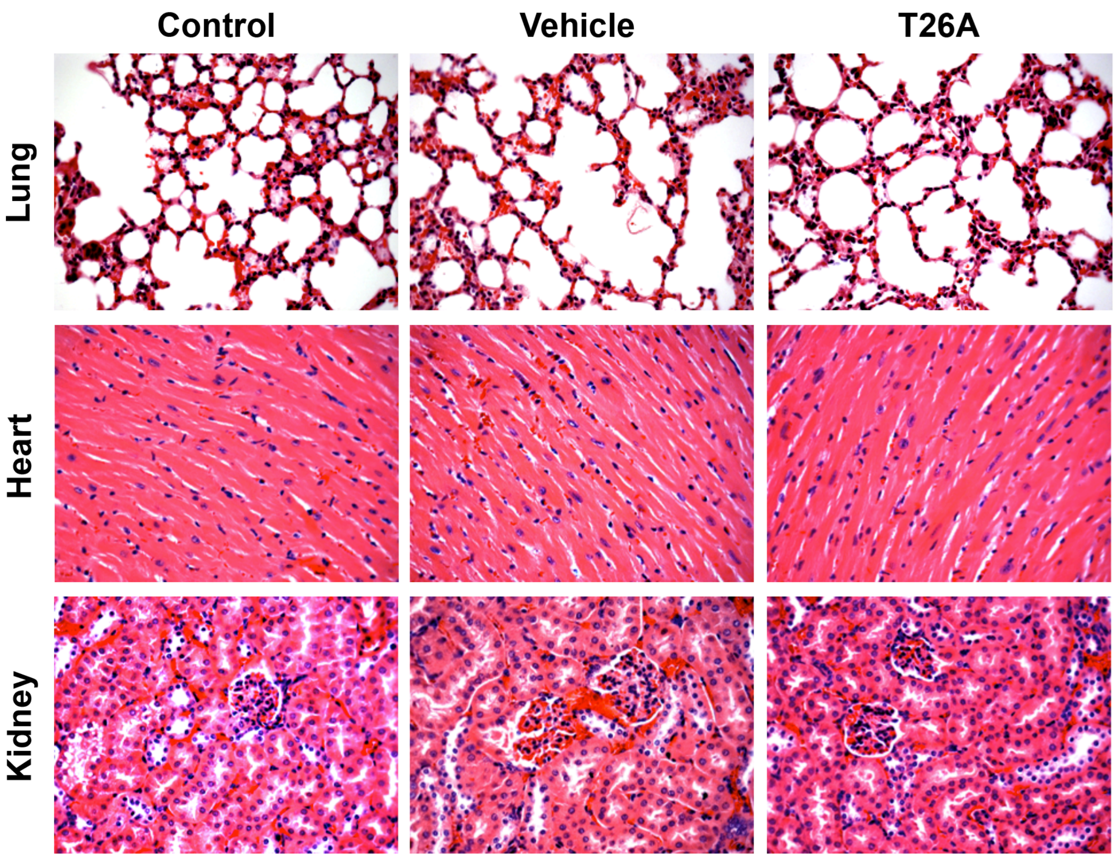

S3 Fig. Histology of lung, heart and kidney. Mice received regular food and water (Ctl), water containing vehicle (2% DMSO + 2% cremophor), or water containing 2 mM T26A for 16 days.
